# A Race against Time: Reduced Azithromycin Susceptibility in *Salmonella enterica* Serovar Typhi in Pakistan

**DOI:** 10.1128/mSphere.00215-20

**Published:** 2020-07-22

**Authors:** Junaid Iqbal, Irum F. Dehraj, Megan E. Carey, Zoe A. Dyson, Denise Garrett, Jessica C. Seidman, Furqan Kabir, Senjuti Saha, Stephen Baker, Farah N. Qamar

**Affiliations:** a Department of Paediatrics and Child Health, Aga Khan University, Karachi, Pakistan; b Cambridge Institute of Therapeutic Immunology and Infectious Disease, Department of Medicine, University of Cambridge, Cambridge, United Kingdom; c Department of Infectious Diseases, Central Clinical School, Monash University, Melbourne, Victoria, Australia; d London School of Hygiene and Tropical Medicine, London, United Kingdom; e Sabin Vaccine Institute, Washington, DC, USA; f Child Health Research Foundation, Dhaka, Bangladesh; JMI Laboratories

**Keywords:** *Salmonella* Typhi, typhoid fever, antimicrobial resistance, azithromycin higher MIC, Pakistan

## Abstract

Antimicrobial resistance is an ongoing issue in the treatment of typhoid fever. Resistance to first-line antimicrobials and extensively drug resistant (XDR) *Salmonella* Typhi isolates in Pakistan have left azithromycin as the only remaining effective oral treatment. Here, we report the emergence of organisms with a single point mutation in *acrB* gene, implicated in azithromycin resistance, in a *S.* Typhi isolate from Pakistan. The isolation of this organism is worrisome and highlights the significance of the introduction of typhoid conjugate vaccine in South Asia.

**IMPORTANCE** The emergence of XDR *Salmonella* Typhi in Pakistan has left azithromycin as the only viable oral treatment option. Here, we report the detection of an azithromycin resistance-associated mutation in one *S.* Typhi isolate. This finding is important because any possible spread of azithromycin resistance in *S.* Typhi isolates would make it nearly impossible to treat in outpatient settings due to the need of injectable antibiotics. Our findings also signify the importance of introduction of typhoid conjugate vaccine in regions of endemicity such as Pakistan.

## Abstract (precis)

The emergence of XDR *Salmonella* Typhi in Pakistan has left azithromycin as the only viable oral treatment option. Here, we report the detection of an azithromycin resistance-associated mutation in one *S.* Typhi isolate. This finding is important because any possible spread of azithromycin resistance in *S.* Typhi isolates would make it nearly impossible to treat in outpatient settings due to the need of injectable antibiotics. Our findings also signify the importance of introduction of typhoid conjugate vaccine in regions of endemicity such as Pakistan.

## OBSERVATION

Typhoid fever, the disease caused by the bacterium *Salmonella* Typhi, is responsible for an estimated 11.8 million infections and 128,200 deaths annually worldwide (1). *S*. Typhi is a human-restricted pathogen that is transmitted via the fecal-oral route. Typhoid mortality ranged from 10–30% of cases in the preantimicrobial era (2), but when treated with effective antimicrobials, typhoid has a case fatality rate of <1% (3). The rise of multidrug resistance (MDR) in the 1990s (4), followed by fluoroquinolone resistance (5), resulted in limited treatment options. The emergence and spread of an extensively drug-resistant (XDR) *S*. Typhi variant in Pakistan (6, 7), which is resistant to chloramphenicol, ampicillin, co-trimoxazole, streptomycin, fluoroquinolones, and third-generation cephalosporins, has left azithromycin as only realistic option for typhoid treatment in Pakistan (8). The recent report of azithromycin-resistant *S*. Typhi in Bangladesh highlights the issues associated with the reliance on this drug and signals the potential of untreatable typhoid (9).

Typhoid is notifiable in Pakistan, and the Aga Khan University has conducted standardized prospective facility and laboratory-based blood culture surveillance in outpatient and inpatient wards at Aga Khan University Hospital and Kharadar General Hospital between September 2016 and September 2019 through the Surveillance for Enteric fever in Asia Project (SEAP). These hospitals serve ∼30 million people, including densely populated informal urban settlements. Subjects presenting to outpatient clinics living in predefined catchment areas with three consecutive days of fever for whom a study clinician recommended a blood culture were enrolled. Inpatients with clinical suspicion of typhoid or with nontraumatic ileal perforation were also enrolled. After blood culture, serologically confirmed *S*. Typhi isolates were subjected to antimicrobial susceptibility testing against azithromycin, ampicillin, co-trimoxazole, chloramphenicol, ciprofloxacin, levofloxacin, ceftriaxone, cefepime, cefixime, and ceftazidime by disk diffusion; resistant organisms (according to CLSI guidelines) were confirmed by Etest (bioMérieux, France) (10).

Between the specified dates, 10,080 patients were enrolled in SEAP in Karachi; 2,104 had a positive blood culture for *S.* Typhi, and 139 had a positive blood culture for *S.* Paratyphi A. Six *S*. Typhi isolates exhibited potential azithromycin resistance by disc diffusion (diameter ≤ 12 mm). Upon MIC testing, one failed to revive, four isolates had azithromycin MICs ranging between 1 and 2 μg/ml and one S. Typhi isolate had an MIC of 12 μg/ml (CLSI susceptibility breakpoint ≤ 16 μg/ml) (10). This places this isolate at the upper range of the wild-type azithromycin susceptibility distribution, with additional resistance to chloramphenicol, fluoroquinolones, and co-trimoxazole, but it was susceptible to third-generation cephalosporins.

We aimed to investigate the genetic basis of the higher azithromycin MIC and place this organism into phylogenetic context with contemporaneous *S.* Typhi through whole-genome sequencing (WGS). Genomic DNA was extracted and subjected to WGS on a Hiseq2500 (Illumina, San Diego, CA) to generate 125-bp paired-end reads. The resulting sequence data were mapped against the CT18 reference sequence (accession no. AL513382) using the RedDog mapping pipeline to identify single-nucleotide variants (SNVs) and to confirm the *S.* Typhi genomes were within H58 lineage I (4.3.1.1) (7, 9, 11–19). (https://github.com/katholt/genotyphi). After removing repetitive sequences and recombination (20), we generated a final alignment 7,661 chromosomal SNVs for 664 isolates (see Table S1 in the supplemental material). Maximum-likelihood phylogenetic trees were inferred from the chromosomal SNV alignments with RAxML (v8.2.9) (21) and visualized in Microreact (22) (https://microreact.org/project/8FjPCdisk) and the ggtree package in R (23). SRST2 (24) was used with ARGannot (25) and PlasmidFinder (26) to identify antimicrobial resistance genes and plasmid replicons, respectively. Mutations in *gyrA*, and *parC*, as well as the R717Q mutation in *acrB*, were detected using GenoTyphi (https://github.com/katholt/genotyphi).

10.1128/mSphere.00215-20.1 TABLE S1 Details of *n* = 664 sequences used in the inference of a H58 lineage I (genotype 4.3.1.1) phylogenetic tree. Accession numbers, source, and year of isolation data for *n* = 664 S. Typhi whole-genome sequences analyzed in this study are presented. Download Table S1, XLSX file, 0.09 MB. Copyright © 2020 Iqbal et al. 2020 Iqbal et al. This content is distributed under the terms of the Creative Commons Attribution 4.0 International license.

**FIG 1:**
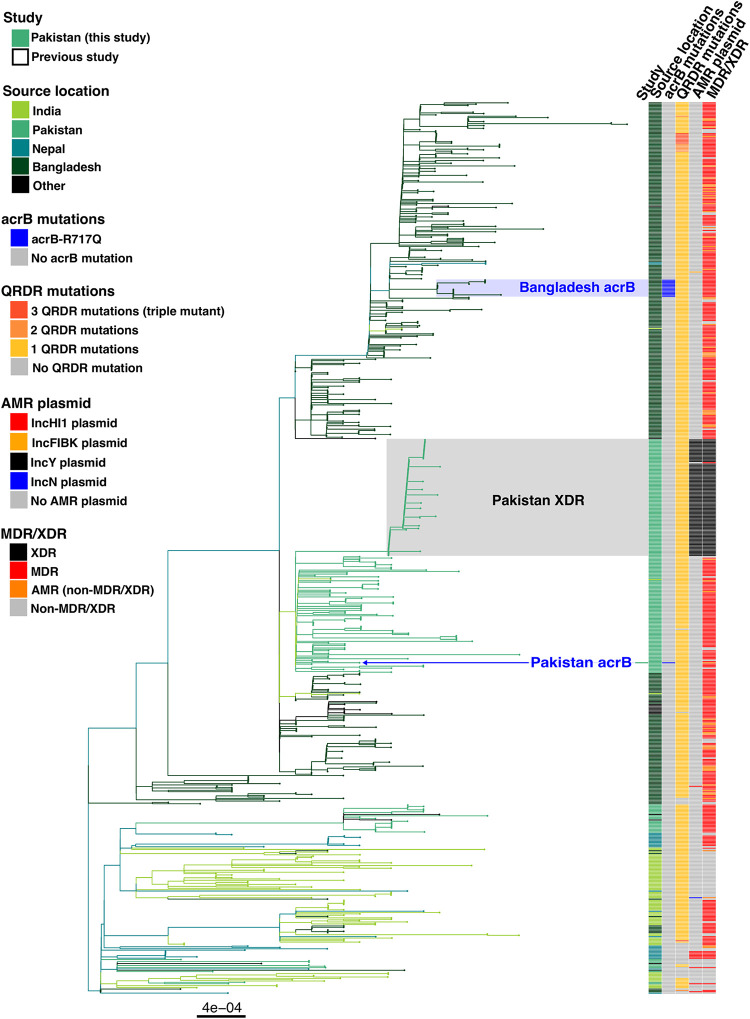
South Asian H58 lineage I (genotype 4.3.1.1) phylogenetic tree (*n* = 664 genomes). Branches are colored by source country according to the inset legend and first color bar. The second color bar indicates genomes containing the *acrB*-R717Q mutation. The third color bar indicates mutations in the quinolone resistance determining region (QRDR) of genes *gyrA*, and *parC*. The final color bar indicates MDR and XDR sequences.

This higher azithromycin MIC *S.* Typhi isolate (MIC of 12 μg/ml), was typed as genotype 4.3.1.1 (H58 lineage I), which is the same sublineage at the XDR clade circulating in Pakistan. The organism additionally had single mutation in *gyrA* (S83F), resulting in reduced fluoroquinolone susceptibility. The apparent mechanism of higher MIC against azithromycin was an R717Q mutation in the gene encoding AcrB, a mutation identical to the recently described azithromycin resistant (MIC of ≥32 μg/ml) *S*. Typhi 4.3.1.1 in Bangladesh (9). The identification of this mutation in *S*. Typhi in Pakistan raises the possibilities that this was either a *de novo* mutation in the Pakistan-specific 4.3.1.1 cluster or an organism that was part of larger, internationally disseminating, azithromycin-resistant clone. To determine which was more likely, we used a collection of 663 South Asian 4.3.1.1 (H58 lineage I) sequences to contextualize *S.* Typhi isolate FQ2181 (7, 9, 11–19). The resulting phylogenetic tree demonstrated that this was a spontaneous mutation which emerged in Pakistan, since it was distantly related (relative within H58 lineage I) to the organisms with *acrB* mutations in Bangladesh, and independent of the proximal XDR sublineage (Fig. 1).

Typically, the isolation of a single *S*. Typhi exhibiting resistance to the primary treatment would not be a major cause for concern. However, this isolate demonstrates an additional, independent acquisition of the same mutation that has been observed in Bangladesh (9). Given the reliance of azithromycin for the treatment of typhoid and other bacterial infections and the “fluoroquinolone experience,” we predict that we are likely to see more of these homoplasies arising. It is too early to predict how these particular organisms may spread, and it is encouraging that these mutations have not yet been reported in XDR *S*. Typhi. However, given the nature of these mutations, one could arise in XDR *S*. Typhi, and/or the XDR plasmid may be mobilized into an azithromycin-resistant lineage.

Pakistan has initiated a nationwide typhoid conjugate vaccine (TCV) rollout program, which began with a mass vaccination in Sindh province in November 2019 (27). Now, there is in a race against time in the prevention of untreatable typhoid fever. With one World Health Organization prequalified manufacturer of TCV supplying vaccine for Gavi-eligible countries and several additional manufacturers in late-stage clinical development (28), there is reason to be optimistic about typhoid control. However, the vaccine is not yet available in all countries of endemicity, and effective treatment is still paramount for typhoid control. Consequently, we need to progress with additional intervention strategies and not overlook that antimicrobials have a substantial impact on typhoid disease control. In addition, as part of this sustained effort, we need to continue to track phenotypic and genotypic antimicrobial resistance in *S*. Typhi to inform best practices for antimicrobial prescribing and the impact of TCV implementation.
